# Interim Evaluation of the Project P.A.T.H.S.: Findings Based on Different Datasets

**DOI:** 10.1100/2012/132826

**Published:** 2012-05-22

**Authors:** Daniel T. L. Shek, Lu Yu

**Affiliations:** ^1^Department of Applied Social Sciences, The Hong Kong Polytechnic University, Hong Kong; ^2^Public Policy Research Institute, The Hong Kong Polytechnic University, Hong Kong; ^3^Department of Social Work, East China Normal University, Shanghai 200062, China; ^4^Kiang Wu Nursing College of Macau, Macau; ^5^Division of Adolescent Medicine, Department of Pediatrics, Kentucky Children's Hospital, University of Kentucky College of Medicine, Lexington, KY 40506, USA

## Abstract

Interim evaluation studies were carried out in order to examine the implementation details of the Tier 1 Program of the Project P.A.T.H.S. (Positive Adolescent Training through Holistic Social Programmes) in Hong Kong. Quantitative results of the interim evaluation findings based on eight datasets collected from 2006 to 2009 are reported in this paper. Three hundred and seventy-eight schools were randomly selected to provide information on the implementation details of the program via face-to-face interviews, telephone interviews, and self-completed questionnaires. Results showed that a majority of the workers perceived that the students had positive responses to the program and the program was helpful to the students. In conjunction with other process evaluation findings, the present study suggests that the implementation quality of the Tier 1 Program of the Project P.A.T.H.S. is high. The present study also provides support for the effectiveness of the Tier 1 Program of the Project P.A.T.H.S. in Hong Kong.

## 1. Introduction

In the positive youth development literature, many researchers argue that building developmental assets in adolescents is critical to the promotion of adolescent health [1, 2]. In addition, there are views emphasizing the importance of holistic youth development, including personal, psychological, social, and spiritual domains [3]. With specific reference to the Chinese culture, while there is a strong emphasis on academic excellence in adolescents, the importance of holistic youth development is not seriously considered by Chinese parents [4]. Furthermore, there are research findings showing that adolescents in Hong Kong face high levels of stress in different psychosocial domains [5]. Against this background, how to promote holistic development in Chinese adolescents and help them to cope with life stresses is an important issue to be considered by professionals working with youth.

To promote holistic adolescent development, The Hong Kong Jockey Club Charities Trust earmarked HK$400 million for a positive youth development program entitled “P.A.T.H.S. to Adulthood: A Jockey Club Youth Enhancement Scheme” for junior secondary school students (secondary 1 to 3 students) in Hong Kong. The word “P.A.T.H.S.” denotes Positive Adolescent Training through Holistic Social Programmes. The project consists of two tiers of programs. While the Tier 1 Program is a universal program aimed at all secondary school students in Hong Kong, the Tier 2 Program targets around one-fifth of the students with greater psychosocial needs. The focus of the present study was to report evaluative findings on the Tier 1 Program.

There are two implementation phases in this project—the experimental implementation phase and the full implementation phase. For the experimental implementation phase (January 2005 to August 2008), 52 secondary schools were invited to participate in the project with the objectives of accumulating experience in program implementation and familiarizing the front-line workers with the program design and philosophy. The full implementation phase started in January 2006. In the 2006/2007 school year, the programs were implemented on a full scale at the secondary 1 level. In the 2007/2008 school year, the programs were implemented at the secondary 1 and 2 levels. In the 2008/09 school year, the programs were implemented at the Secondary 1, 2, and 3 levels [6, 7]. Because of the positive outcomes of the project, an extension phase with another cycle (2009–2012) was approved by the Trust, with an additional earmarked grant of HK$350 million.

In the Tier 1 Program, students in secondary 1 to 3 participate, normally with 20 h of training in the school year at each grade. The research team has developed a set of curriculum manuals, which includes curriculum materials based on 15 positive youth development constructs identified from the existing successful positive youth development programs: bonding, resilience, social competence, emotional competence, cognitive competence, behavioral competence, moral competence, self-determination, spirituality, self-efficacy, clear and positive identity, beliefs in the future, recognition for positive behavior, prosocial involvement, and prosocial norms [8]. In each grade, 40 teaching units, each of 30-minute duration, have been designed based on the theoretical framework of positive youth development constructs, relevant research findings, and existing programs in both local and foreign contexts.

Because adolescent development is influenced by the interactions between young people and their surrounding environment, the ecological perspective was adopted in the Project P.A.T.H.S. when designing the teaching units that are intended to cultivate students' development in five different domains—individual, family, peer, school, and society. For example, some units in the secondary 1 curriculum were designed to promote students' relationships with their teachers and classmates, which helps the secondary 1 students to adapt to their new school life. Moreover, because there are worrying trends and phenomena related to the development of adolescents in Hong Kong, such as mental health problems, abuse of psychotropic substances, adolescent suicide, school violence, and a drop in family solidarity [6], some teaching units were designed to tackle current youth issues, such as (1) mental health problems, for example, depressed mood and Internet addiction; (2) substance abuse and smoking; (3) heterosexual relationships; (4) materialism. Furthermore, various kinds of teaching materials (e.g., student worksheets, PowerPoint presentations, and soundtracks) were developed in order to facilitate the transmission of the learning targets of the teaching units.

To evaluate the effectiveness of the Project P.A.T.H.S., several mechanisms involving different stakeholders have been used with multiple types of data collected. These include objective outcome evaluation, subjective outcome evaluation, qualitative evaluation, management information collected from the cowalker scheme, process evaluation, interim evaluation, and evaluation based on personal construct psychology. Among these approaches, process evaluation and interim evaluation constitute an indispensable part of program evaluation that helps researchers to monitor program adherence, create an infrastructure that supports the project, evaluate how effectively that process functions, and assess changes in skills, attitudes, and knowledge of the participants and program implementers [9, 10]. Through such evaluative methods, the implementation process of the program can be adequately understood and monitored, and thus the quality of implementation service is ensured throughout each stage.

Nevertheless, in the context of evaluation of positive youth development programs, a survey of the literature shows that findings on program implementation quality are rarely reported [9, 11, 12]. Since most evaluation studies have focused primarily on objective outcome evaluation, recent process evaluation studies represent a concerted effort to fill the gap [4, 5, 13, 14]. Shek et al. [13] pointed out several fatal consequences of overlooking the quality of the implementation of a program. First, the “black box” approach (i.e., focus on the input and output alone) would make it difficult to understand the process of the program success or failure. Second, the lack of process evaluation would prevent the program developers from looking at the strengths and weaknesses of the programs developed. Third, the developers and implementers could not effectively decide how the program would be more effective if offered again. Finally, without process evaluation, program developers have to wait until the outcome data are collected if they wish to refine the program.

In the same vein, there are several arguments for conducting process evaluation [15]. First, process evaluation can tell the program developers whether a Type III error (i.e., existence or nonexistence of program outcomes because of occurrence of activities different from those intended by the program developers) has occurred. Second, fidelity in program implementation can be promoted by feedback collected in the implementation process. Third, process evaluation can help program developers to understand whether and to what extent the intended targets receive the program. Fourth, process evaluation can help to identify factors that contribute to program success or failure. Finally, program developers can use process evaluation findings to understand how the developed program can be successfully implemented in human organizations and communities that are always complex in nature.

Weinbach [16] provided two further reasons to support conducting process evaluation. First, it can provide some valuable insights about a program. Second, it examines a program somewhat broadly, much like a “systems analysis” to examine how the program works overall. In its broad sense, the central research question of a process evaluation is “What happened and why did it happen?” The implicit research hypothesis is “Something happened that affected the program's ability to achieve its outcomes.” Other specific research questions for a process evaluation can also be used, such as “How did the program come into existence in the first place? What changes occurred over time that were unplanned and inconsistent with the program model? What should be done differently if a similar program is to be undertaken?” [16, page 168].

There are many forms of process evaluation studies. One possible form is to send observers to monitor the actual implementation of the program, where the implementation details, such as fidelity, student involvement, and implementer's skills, are rated. Another possibility is to collect feedback about the implementation process from program workers and participants so that some interim assessments can be carried out to obtain more information about the implementation process. Interim evaluation serves at least four purposes. First, it helps the program developers to identify whether there are any problems in implementation so that corrective measures or adjustments to the program can be stepped up where appropriate. Second, interim evaluation as a potential asset to program management provides valuable information on the progress of the implementation. Third, interim evaluation serves as a gesture of concern and encouragement, and it serves as a bridge between the program developers and the program implementers. Finally, the instant feedback of the interim evaluation findings to the program implementers can help to boost the morale and ownership of the program implementers.

In evaluating the Project P.A.T.H.S. in Hong Kong, while process evaluation has been conducted through systematic observation on the program implementation details, interim evaluation in several cohorts has also been carried out during the program implementation process. Based on the program implementers' comments regarding the whole process of program implementation, more understandings of the reactions of the participants and workers to the program are gained. Via both face-to-face interviews and telephone interviews, interim evaluation information in the following areas is collected: (1) program workers' perceptions of the responses of the participants to the program, (2) experiences of the program workers delivering the program, (3) program implementers' perceived helpfulness of the program, (4) program implementers' perceived positive aspects of the program, (5) aspects of the program that require improvement, (6) difficulties encountered during program implementation, and (7) overall evaluation of the program. Several interim evaluation studies on the Project P.A.T.H.S.. have been published in peer-reviewed, international journals. Results show that both the program implementers and participants had positive comments on the program, although the workers also encountered problems and difficulties in the implementation process [17–19]. In this paper, the several cohorts of data were merged, and integrative analyses were carried out in order to understand the implementation details. It is expected that the present study will provide a general picture about the implementation of the Project P.A.T.H.S. over the past 4 years based on interim evaluation.

## 2. Methods

### 2.1. Participants and Procedures

From 2005 to 2009, the total number of schools that participated in the Project P.A.T.H.S. was 244. Among them, 46.27% of the respondent schools adopted the full program (i.e., 20-hour program involving 40 units), whereas 53.73% of the respondent schools adopted the core program (i.e., 10-hour program involving 20 units).

Among all the participating schools, 236 schools that joined the 20-hour full program and 167 schools that joined the 10-hour core program were randomly selected to participate in the interim evaluation study. A total of 265 teachers and 178 social workers were invited to participate in face-to-face interviews on a voluntary basis during school visits. If the respondents were not available for the face-to-face interviews during the school visits, they were invited to participate in telephone interviews. Otherwise, they were asked to complete a self-administered questionnaire and return it to the research team via e-mail or fax. The random sampling method increased the validity of the findings. Descriptions of the datasets collected over the 4 years can be seen in Table 1.

### 2.2. Instruments

In the 2005/2006 school year, a self-constructed, semistructured interview guide with six open-ended questions was used to collect information on the program implementation process. In the 2006/2007 to 2008/2009 school years, a modified, self-constructed, semistructured interview guide was developed and used with five closed-ended questions as follows.

- Question 1: what do you think about students' involvement in the program? (a 4-point scale).
- Question 2: do you think the students like the program? (a 4-point scale).
- Question 3: to what degree do you think the Tier 1 Program is helpful to students? (a 5-point scale).
- Question 4: do you like this program? (a 4-point scale).
- Question 5: your overall satisfaction to the program is? (a 6-point scale).

Seven open-ended questions were also used to collect information on the program implementation process. The open-ended questions were as follows.

- Question 1: what are the responses of the students to this program?
- Question 2: do you think this program is beneficial to the students? If yes, what are the benefits?
- Question 3: what are the good aspects of the program?
- Question 4: which areas of the program require improvement?
- Question 5: have you encountered any difficulties during the program implementation process? If yes, what problems have you encountered?
- Question 6: what are your perceptions of the “Cowalker scheme”?
- Question 7: do you have other opinions?

The qualitative data were analyzed by two trained research assistants. For the quantitative data (closed-ended questions), frequencies and percentages of responses were calculated.

## 3. Results

Since the instrument used for data collection in the 2005/2006 school year was different from others, no data collected in the 2005/2006 school year was presented. In the present paper, only the quantitative results of the interim evaluation in the 2006–2009 school years are reported. First, 92.86% of 378 respondent schools reported that students were involved in the program, which included 94.17, 94.48, and 89.38% of the workers implementing the secondary 1, 2, and 3 programs, respectively (Table 2). Second, for perceived students' liking of the curriculum, positive responses were found in 93.33% of the secondary 1 instructors, 96.55% of the secondary 2 instructors, and 91.15% of the secondary 3 instructors, indicating that, on average, 93.92% of the program implementers perceived that students liked the curriculum (Table 3). Third, concerning the perceived benefits of the program to the students, 95.50% of the respondents regarded the Tier 1 Program as helpful to the students (Table 4), including 95.00% of the secondary 1 workers, 93.79% of the secondary 2 workers, and 98.23% of the secondary 3 workers.

Table 5 presents the perceived liking of instructors toward the program and shows that 87.83% of the respondents indicated positive results. By grade, 85.00% of the secondary 1 implementers, 89.66% of the secondary 2 implementers, and 88.50% of the secondary 3 implementers agreed that they liked the program. As shown in Table 6, an average of 94.75% of the program implementers were satisfied with the program, including 90.00% of the secondary 1 workers, 95.18% of the secondary 2 workers, and 98.23% of the secondary 3 workers.

## 4. Discussion

Based on several datasets collected in the experimental implementation phase and the full Implementation phase, the present paper integrates, analyzes, and interprets interim evaluation findings of the Tier 1 Program of the Project P.A.T.H.S. over time. There are several unique features of this study. First, a large sample involving a large number of teachers and social workers was used in this study. Second, data collected over different cohorts were utilized. Third, in view of the paucity of interim evaluation findings in both western and Chinese contexts, the present study is a pioneering study in the literature. Actually, this is the first known scientific interim evaluation study based on a series of evaluation studies in the Chinese evaluation literature.

Several phenomena can be highlighted from the present study. First, the program implementers perceived that the students were involved in the program. This finding is consistent with the previous findings on process evaluation where students were observed to be highly involved in the Tier 1 Program. Because the activities in the Project P.A.T.H.S. strongly encourage student participation and the implementers are expected to teach in an interactive manner, it is not surprising to find that the students were highly involved in the program. This observation strongly suggests that when designing positive youth development programs, how to promote student involvement is an important consideration.

The findings also show that both students and the program implementers indicated that they liked the program. This observation is generally consistent with the previous subjective outcome evaluation findings where both program participants and program implementers indicated that they liked the Tier 1 Program. As indicated, the interactive and participative nature of the program is quite unlike the regular subjects in the formal curriculum. The findings are also consistent with the qualitative evaluation findings that the program was perceived in a positive manner by the program participants and implementers. Taken as a whole, the present findings are consistent with the subjective outcome and qualitative evaluation findings based on different stakeholders.

Finally, both the program participants and implementers perceived the Tier 1 Program to be beneficial to the program participants. This observation also echoes the subjective outcome and qualitative findings reported previously. In addition, this observation is in line with the objective outcome evaluation findings. For example, Shek and Sun [20] reported findings on the objective outcome evaluation of the project. At the third year of the full implementation phase, 19 experimental schools (*n* = 3, 170 students) and 24 control schools (*n* = 3, 808 students) participated in a randomized group trial. Utilizing the 6-wave longitudinal data, analyses of covariance and linear mixed models controlling for differences between the two groups in terms of wave 1 pretest scores revealed that participants in the experimental schools showed significantly better developmental outcomes than did participants in the control schools at posttest (wave 6) based on different indicators of positive youth development derived from the Chinese Positive Youth Development Scale and other measures. Students in the experimental schools also displayed a lower level of intention to engage in problem behavior and better school adjustment than did students in the control schools. Similarly, differences between experimental participants who perceived the program to be beneficial and control participants were found. Similar analyses based on linear mixed models via the Statistical Package for Social Sciences showed that participants in the experimental schools displayed better positive youth development than did participants in the control schools in terms of different positive youth development indicators, including positive self-identity, prosocial behavior, and general positive youth development attributes [21], and that the experimental participants also showed lower levels of various problem behaviors [22].

When a psychosocial intervention program is designed, one basic question is whether the developed program is effective. In the evaluation literature, many strategies have been proposed to evaluate the effectiveness of a psychosocial intervention program, such as objective outcome evaluation and subjective outcome evaluation [23]. While the outcomes of a program are important to consider, it is equally important to appreciate the fact that the outcomes of an intervention program are contingent on the quality of program implementation. As such, it is crucial to understand the quality of the program implementation process via process evaluation and interim evaluation [15]. With specific reference to interim evaluation, several sets of functions can be identified. First, interim evaluation examines the delivery of programs, including ascertaining the nature of the program and whether it is implemented in the intended manner. Second, it reviews the relevance of the program in relation to its objectives. Third, interim evaluation can assess short-term impact in the midpoint of program implementation. Fourth, it examines management performance. Finally, interim evaluation identifies potential problems in the program implementation and contributes to the improvement and adjustment of the program [24, 25]. Despite its importance, a survey of the literature shows that evaluation studies on adolescent prevention programs have been based primarily on objective outcome evaluation, and there are few studies that have examined the implementation process [11, 12, 26]. Obviously, the present integrative study provides some useful findings on the implementation quality of the Project P.A.T.H.S. in Hong Kong.

Despite the positive evaluation findings, there are two limitations of the study that should be taken into account when the findings are interpreted. First, because the findings are based on the subjective perceptions of the program implementers only, subjective biases involved must be considered. Second, from the qualitative findings of interim evaluation previously reported, some problems encountered in the implementation process and recommendations for improvement were noted. Notwithstanding these limitations, together with other evaluation findings [27], the interim evaluation findings across studies generally suggest that the program is positively perceived by the program participants and implementers, and different stakeholders regard the program to be beneficial to the program participants.

According to Meyer et al. [28], the development of a feedback loop from the participants and workers regarding the program implementation is important for program refinement. As Gomby and Larson [29] described, “process evaluation focuses on what services were provided to whom and how. Its purpose is to describe how the program was implemented—who was involved and what problems were experienced. A process evaluation is useful for monitoring program implementation, for identifying changes to make the program operate as planned, and generally, for program improvement” [29, page 71]. In the western context, interim evaluation as a form of process evaluation has been carried out in different human services contexts [30–33]. The current integrative study adds to the existing literature on the importance of interim evaluation in the context of positive youth development programs.

## Figures and Tables

**Table 1:** Different datasets used in the integrative study.

	S1				S2			S3	
	2005/06	2006/07	2007/08	2008/09	2006/07	2007/08	2008/09	2007/08	2008/09
	EIP	FIP	FIP*	FIP	EIP	FIP	FIP	EIP	FIP
Total schools that joined the project P.A.T.H.S.	52	207	213	197	49	196	198	48	167
(i) 10-hour program	23	95	108	104	27	113	110	29	104
(ii) 20-hour program	29	112	105	93	22	83	88	19	63
Total schools joined this study	25	100	NA	20	25	100	20	25	88
(i) 10-hour program	10	30	NA	5	11	39	6	13	53
(ii) 20-hour program	15	70	NA	15	14	61	14	12	35
Total respondents	28	111	NA	21	32	114	20	29	88
(i) Teachers	25	66	NA	12	23	64	14	11	50
(ii) Social workers	3	45	NA	9	9	50	6	18	38

Note: Data based on consolidation table. S1: secondary 1 level; S2: secondary 2 level; S3: secondary 3 level; EIP: experimental implementation phase; FIP: full implementation phase; NA: not available.

*For the 2007/08 school year, no data were collected at the S1 level.

**Table 2:** Degree of student involvement perceived by the program implementers.

		Negative response			Positive response			No response	All
		Totally not involved	Not involved	Total	Involved	Totally involved	Total		
S1	*n*	0	6	6	102	11	113	1	120
	Percentage	0%	5.00%	5.00%	85.00%	9.17%	94.17%	0.83%	100%
S2	*n*	0	7	7	129	8	137	1	145
	Percentage	0%	4.83%	4.83%	88.97%	5.52%	94.48%	0.69%	100%
S3	*n*	0	10	10	94	7	101	2	113
	Percentage	0%	8.85%	8.85%	83.19%	6.19%	89.38%	1.77%	100%

Total	*n*	0	23	23	325	26	351	4	378
	Percentage	0%	6.08%	6.08%	85.98%	6.88%	92.86%	1.06%	100%

**Table 3:** Degree of students' liking of the program perceived by the program implementers.

		Negative response			Positive response			No response	All
		Strongly dislike	Dislike	Total	Like	Strongly like	Total		
S1	*n*	0	5	5	108	4	112	3	120
	Percentage	0%	4.17%	4.17%	90.00%	3.33%	93.33%	2.50%	100%
S2	*n*	0	4	4	137	3	140	1	145
	Percentage	0%	2.76%	2.76%	94.48%	2.07%	96.55%	0.69%	100%
S3	*n*	0	8	8	100	3	103	2	113
	Percentage	0%	7.08%	7.08%	88.50%	2.65%	91.15%	1.77%	100%

Total	*n*	0	17	17	345	10	355	6	378
	Percentage	0%	4.50%	4.50%	91.27%	2.65%	93.92%	1.59%	100%

**Table 4:** Degree of perceived helpfulness of the curriculum to the student perceived by the program implementers.

		Negative response			Positive response				No response	All
		Unhelpful	Not very helpful	Total	Slightly helpful	Helpful	Very helpful	Total		
S1	*n*	0	4	4	57	54	3	114	2	120
	Percentage	0%	3.33%	3.33%	47.50%	45.00%	2.50%	95.00%	1.67%	100%
S2	*n*	0	7	7	74	56	6	136	2	145
	Percentage	0%	4.83%	4.83%	51.03%	38.62%	4.14%	93.79%	1.38%	100%
S3	*n*	0	1	1	58	49	4	111	1	113
	Percentage	0%	0.88%	0.88%	51.33%	43.36%	3.54%	98.23%	0.88%	100%

Total	*n*	0	12	12	189	159	13	361	5	378
	Percentage	0%	3.17%	3.17%	50%	42.06%	3.44%	95.50%	1.32%	100%

**Table 5:** Degree of liking of the curriculum by the program implementers.

		Negative response			Positive response			No response	All
		Strongly dislike	Dislike	Total	Like	Strongly like	Total		
S1	*n*	0	3	3	95	7	102	15	120
	Percentage	0%	2.50%	2.50%	79.17%	5.83%	85.00%	12.50%	100%
S2	*n*	0	1	1	116	14	130	14	145
	Percentage	0%	0.69%	0.69%	80.00%	9.66%	89.66%	9.66%	100%
S3	*n*	0	0	0	91	9	100	13	113
	Percentage	0%	0%	0%	80.53%	7.96%	88.50%	11.50%	100%

Total	*n*	0	4	4	302	30	332	42	378
	Percentage	0%	1.06%	1.06%	79.89%	7.94%	87.83%	11.11%	100%

**Table 6:** Perceived degree of workers' overall satisfaction of the curriculum.

		Negative response				Positive response				No response	All
		Very dissatisfied	Dissatisfied	Slightly dissatisfied	Total	Slightly satisfied	Satisfied	Very satisfied	Total		
S1	*n*	0	1	7	8	26	78	4	108	4	120
	Percentage	0%	0.83%	5.83%	6.66%	21.67%	65.00%	3.33%	90.00%	3.3%	100%
S2	*n*	0	0	7	7	40	96	2	138	0	145
	Percentage	0%	0%	4.83%	4.83%	27.59%	66.21%	1.38%	95.18%	0%	100%
S3	*n*	0	0	2	2	21	88	2	111	0	113
	Percentage	0%	0%	1.77%	1.77%	18.58%	77.88%	1.77%	98.23%	0%	100%

Total	*n*	0	1	16	17	87	262	8	357	4	378
	Percentage	0%	0.26%	4.23%	4.49%	23.02%	69.61%	2.12%	94.75%	1.06%	100%
